# Phase-specific kidney graft failure prediction with machine learning model

**DOI:** 10.3389/frai.2025.1682639

**Published:** 2025-10-02

**Authors:** Amankeldi A. Salybekov, Markus Wolfien, Ainur Yerkos, Zholdas Buribayev, Sumi Hidaka, Shuzo Kobayashi

**Affiliations:** ^1^Kidney Disease and Transplant Center, Shonan Kamakura General Hospital, Kamakura, Japan; ^2^Regenerative Medicine Division, Cell and Gene Therapy Department, Qazaq Institute of Innovative Medicine, Astana, Kazakhstan; ^3^Faculty of Medicine Carl Gustav Carus, Institute for Medical Informatics and Biometry, TUD Dresden University of Technology, Dresden, Germany; ^4^Center for Scalable Data Analytics and Artificial Intelligence (ScaDS.AI), Dresden, Germany; ^5^Department of Computer Science, Al-Farabi Kazakh National University, Almaty, Kazakhstan

**Keywords:** kidney transplantation, graft failure, machine learning, deceased donor, survival prediction

## Abstract

**Background:**

Accurate prediction of kidney graft failure at different phases post-transplantation is critical for timely intervention and long-term allograft preservation. Traditional survival models offer limited capacity for dynamic, time-specific risk estimation. Machine learning (ML) approaches, with their ability to model complex patterns, present a promising alternative.

**Methods:**

This study developed and dynamically evaluated phase-specific ML models to predict kidney graft failure across five post-transplant intervals: 0–3 months, 3–9 months, 9–15 months, 15–39 months, and 39–72 months. Clinically relevant retrospective data from deceased donor kidney transplant recipients were used for training and internal validation, with performance further confirmed on a blinded external validation cohort. Predictive performance was assessed using ROC AUC, F1 score, and G-mean.

**Results:**

The ML models demonstrated varying performance across time intervals. Short-term predictions in the 0–3 month and 3–9 month intervals yielded moderate accuracy (ROC AUC = 0.73 ± 0.07 and 0.72 ± 0.04, respectively). The highest predictive accuracy observed in mid-term or the 9–15-month window (ROC AUC = 0.92 ± 0.02; F1 score = 0.85 ± 0.03), followed by the 15–39-month period (ROC AUC = 0.84 ± 0.04; F1 score = 0.76 ± 0.04). Long-term prediction from 39 to 72 months was more challenging (ROC AUC = 0.70 ± 0.07; F1 score = 0.65 ± 0.06).

**Conclusion:**

Phase-specific ML models offer robust predictive performance for kidney graft failure, particularly in mid-term periods, supporting their integration into dynamic post-transplant surveillance strategies. These models can aid clinicians in identifying high-risk patients and tailoring follow-up protocols to optimize long-term transplant outcomes.

## 1 Introduction

Chronic kidney diseases (CKD) affect an estimated 9.1% of the global population, potentially indicating a trend of increasing prevalence or growing burden of CKD (Bikbov et al., 2020). Of note, this prevalence is very likely an underestimate owing to the lack of early kidney disease detection and screening programs in many parts of the world, which results in large-scale unawareness of the burden and prevalence of earlier stages of CKD. Further progression of CKD commonly ends up with End-stage kidney disease (ESKD) that affects patients’ quality and length of life, representing a large portion of healthcare expenditure for renal replacement therapy or kidney transplantation (Jha et al., 2023; Zhang et al., 2023). The number of people receiving renal replacement therapy exceeds 2.5 million and is projected to double to 5.4 million by 2030 (Bikbov et al., 2020). However, in many countries, there is a shortage of renal replacement and kidney transplantation services, and an estimated 2.3–7.1 million adults have died prematurely from lack of access to this treatment (Mudiayi et al., 2022; Bikbov et al., 2020). Kidney transplantation is one of the most effective methods of treating ESKD.

In clinical graft outcome prediction studies frequently uses the Cox proportional hazards (PH) model to estimate kidney graft survival (Poggio et al., 2021; Huang et al., 2022). The Cox PH model, a classical time-to-event analysis approach, models the hazard function as a function of time and remains widely utilized due to its robustness, reliability, and interpretability for clinicians. However, conventional models have limitations in capturing non-linear relationships and high-dimensional interactions among predictors (Mulugeta et al., 2025). As a result, existing clinical risk scores and Cox-based tools only achieve modest accuracy in predicting graft outcomes (typically with ROC AUC in the 0.60–0.70 range; Naqvi et al., 2021). This moderate performance underlines the need for more powerful prognostic methods to better stratify transplant patients by graft failure risk.

In recent years, machine learning (ML) approaches have gained attention in transplant medicine for their potential to improve predictive performance. ML algorithms can automatically learn complex patterns from large datasets without relying on *a priori* assumptions, which is advantageous given the multifactorial nature of graft failure (Naqvi et al., 2021). Several studies have reported that ML-based models outperform traditional Cox models in discrimination and overall accuracy for graft survival prediction. For example, Naqvi et al. developed ML models on a national transplant registry and achieved area-under-the-curve values of ~0.82 for 1-year and ~0.81 for long-term graft survival, significantly higher than those of earlier risk prediction tools (Naqvi et al., 2021). Another key consideration is that most prognostic models for kidney graft survival is whether traditional or ML-based have been static, using only baseline variables at transplant to predict outcomes far in the future (Huang et al., 2022). In practice, transplant recipients undergo regular follow-up, during which their clinical parameters (e.g., renal function, immunosuppressive levels, etc.) evolve over time. Ignoring these longitudinal changes can limit predictive accuracy (Huang et al., 2022). To address this, dynamic prediction models and updated risk estimates in patient data are necessary. This study aimed to develop and dynamically assess phase-specific machine learning models for the prediction of kidney graft failure across five clinically relevant post-transplant intervals.

## 2 Materials and methods

### 2.1 Data source and justification of study period

This study is predicated upon data derived from the United States National Kidney Transplantation Database (UNOS/OPTN), spanning the years 2015 to 2021. The selection of this specific time frame is justified by several factors. Firstly, the implementation of new clinical guidelines for kidney transplantation in 2015 renders the data from this period particularly relevant for contemporary research (Abramowicz et al., 2015). Secondly, utilizing data from the most recent five-year period facilitates the training of ML models with more representative information, thereby enhancing the accuracy of predictions. For the data analysis, we included pre-operative patients’ and donors’ data (waiting list records), and follow-up data from recipients (Salybekov et al., 2024).

### 2.2 Study cohort and data selection criteria

Selected transplant recipients and donors were between 18 to 80 years old and had undergone a primary kidney transplantation. In our study, we set inclusion and exclusion criteria to minimize potential biases and ensure the analysis was conducted on a cohort most representative of the target patient population (TRIPOD+AI, 2015; von Elm et al., 2007). To ensure our reporting was comprehensive and transparent, we followed the TRIPOD+AI checklist, which is provided in its entirety in Supplementary Table 1. This approach adhered to ethical guidelines for patient data utilization, ensuring privacy and responsible research practices (Salybekov et al., 2025).

Considering the STROBE guidelines, the study design aimed to ensure transparency and completeness in reporting the results (Salybekov et al., 2024; von Elm et al., 2007). Specifically, the following cases were excluded to enhance the precision of our findings:

- Patient deaths unrelated to kidney failure or transplant rejection.
- Pediatric patients.
- Patients from ethnic minority groups.
- Repeated kidney and simultaneous kidney-pancreas transplant recipients were excluded to focus on initial transplantation outcomes and evaluate current treatment effectiveness and complication risk factors.

Prior to imputation, features with more than 30% missing values were removed. This threshold was chosen based on previous research in similar domains (Abdollahzade et al., 2025; Taherkhani et al., 2021; Chang et al., 2019). The remaining missing values were imputed using multiple imputation chained equations (MICE) via the miceforest package. The MICE algorithm is effective for handling missing values up to 50% (Junaid et al., 2025), making it a suitable method for this analysis. Undersampling was conducted to control data imbalance, such as the number of observations in the majority class was reduced to match that of the minority class through RandomUnderSampling. The iteration rate was set to 100 repetitions. It is important to acknowledge that while imputation is necessary for model training, it carries the potential to introduce bias, particularly if some patient information was deliberately omitted (Shadbahr et al., 2023). In our study, imputation was conducted to ensure data completeness for model training purposes, and the potential impact on diagnostic classification was subsequently considered during result interpretation.

The dataset was stratified into five distinct cohorts based on post-transplantation preliminary statistical findings as 0–3, 3–9, 9–15, 15–39, and 39–72 months, respectively. For each cohort, repetitive samples were generated by selecting data from a single randomly selected patient visit within the specified period. Regarding the 15–39 and 39–72 months cohorts, three patient records per subject were included. This multi-stage analytical approach enabled the identification of latent patterns that might have remained undetected in a single time-point analysis. Essentially, this methodology serves as a form of dynamic prediction, allowing for the continuous adjustment of prognostic models in real time by accounting for patient condition fluctuations (Raynaud et al., 2021).

### 2.3 Model development and evaluation

**Figure 1:**
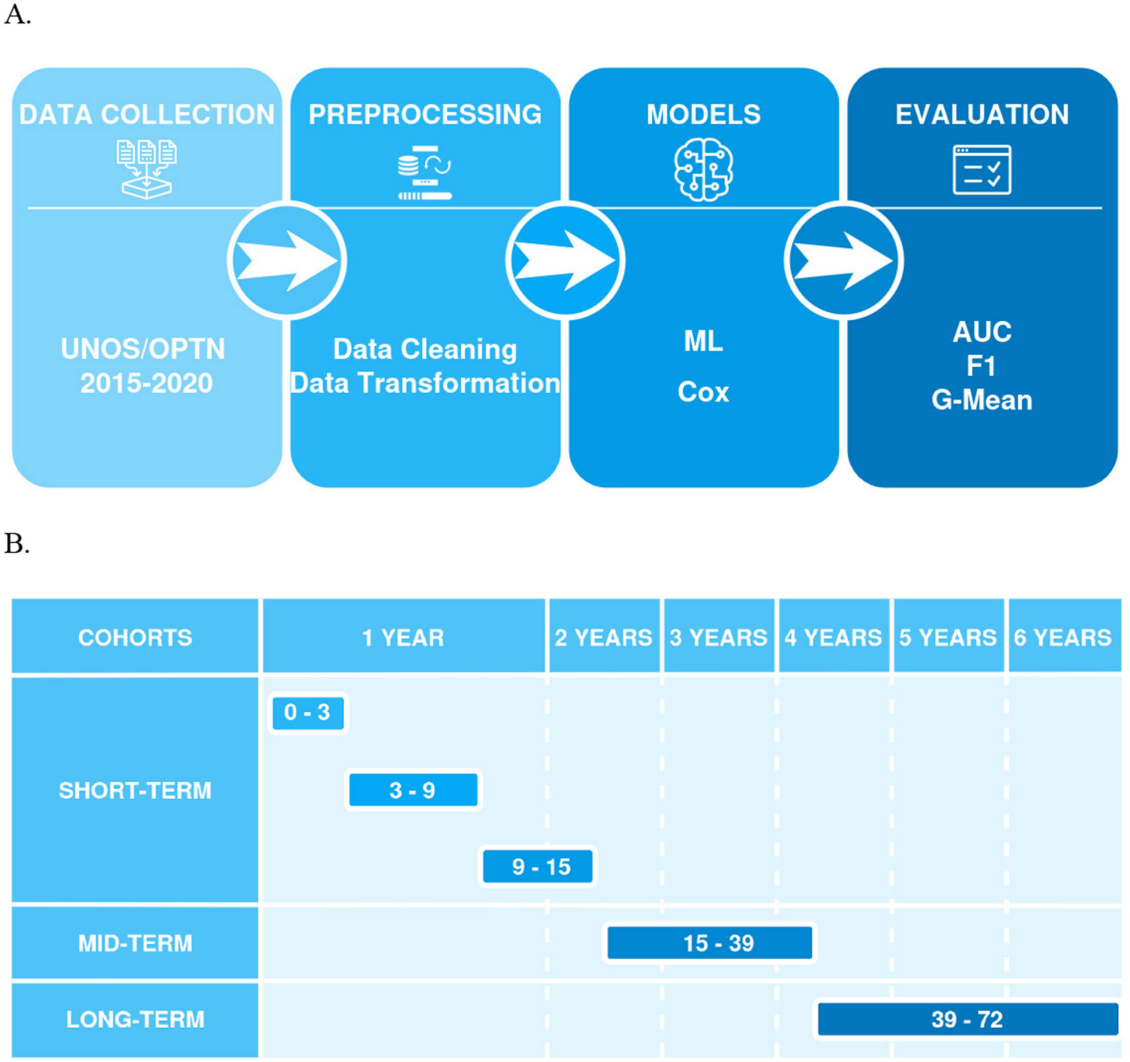
**(A)** Shows a flowchart of a machine learning process, including stages for data collection (UNOS/OPTN 2015–2020), preprocessing (data cleaning and transformation), models (ML, Cox), and evaluation (AUC, F1, G-Mean), connected by arrows indicating the sequence of steps; **(B)** Presents a timeline for model predictions, categorized into cohorts (short-term, mid-term, and long-term) and time intervals in months (0–3, 3–9, 9–15 for short-term, 15–39 for mid-term, and 39–72 for long-term).

In accordance with the procedure outlined in Figure 1, four distinct modeling approaches were investigated. First, standalone ML models were developed through a sequential process. The dataset was initially partitioned into training and testing subsets, with 10% reserved for testing. This ensures unbiased evaluation. The remaining training set underwent stratified 5-fold cross-validation (StratifiedKFold). This process divided the training data into five equally sized “folds,” maintaining class proportions in each. For each iteration, one-fold served as the validation set, while the other four were used for model training. This method provides a reliable performance estimate and helps mitigate overfitting. Subsequently, feature selection was performed on the combined train+validation set using the three commonly used ML algorithms, namely RF (Random Forest), XGB (XGBoost), and LGBM (LightGBM), along with an ensemble model that was subsequently trained to compare the overall performance. These algorithms were implemented in Python using the packages sklearn (v. 1.5.2), xgboost (v. 2.1.2), and lightgbm (v. 4.3.0), respectively. Second, a standalone Cox Proportional Hazards Regression (Cox PHR) model was implemented as a baseline for time-to-event analysis. Model training and optimal hyperparameter tuning were performed using the GridSearchCV approach (González-Castro et al., 2024) with fivefold cross-validation. The mean ROC-AUC served as the primary performance metric, ensuring robust and reliable model evaluation. In addition, F1 and G-Mean scores were employed to assess the overall predictive capability of the trained models.

### 2.4 Statistical methods

To facilitate more in-depth analysis, a Cox proportional hazards regression model was utilized to calculate the concordance index (CI), thereby providing an additional layer of statistical validation. A significance level of 0.05 was adopted for hypothesis testing, which is standard practice in statistical research and ensures robust conclusions regarding the significance of the results. All statistical analyses and ML model development were conducted using Python v. 3.11.5 (Python, 2023).^1^

## 3 Results

### 3.1 Patient demographic data

Clinically relevant data from transplant recipients and donors spanning the period 2015–2020 were employed to develop the predictive model, followed by its evaluation using a test cohort. Additionally, a blinded external validation cohort was utilized to further substantiate the model’s performance. The baseline characteristics of the study cohorts were stratified across five-time intervals: 0–3 months (*N* = 298), 3–9 months (*N* = 362), 9–15 months (*N* = 828), 15–39 months (*N* = 617), and 39–72 months (*N* = 452). The mean age of recipients (R) all in five consequative time period are without statistical significance, ranging from 51 ± 14 to 54 ± 13 years, while the donors (D) showed a slightly younger profile with mean ages between 40 ± 13 and 43 ± 13 years. Body Mass Index for both recipients and donors demonstrated minimal variation across the time points, averaging around 28–29 kg/m^2^. All baseline characteristics of R and D are shown in Table 1.

**Table 1:** Baseline characteristics for the study cohorts.

Name of the features	From 0 to 3 months (*N* = 298)	From 3 months to 9 months (*N* = 362)	From 9 months to 15 months (*N* = 828)	From 15 months to 39 months (*N* = 617)	From 39 months to 72 months (*N* = 452)
Age (R)	52 ± 14	54 ± 13	53 ± 14	52 ± 14	51 ± 14
Age (D) Baseline	42 ± 13	42 ± 14	43 ± 13	41 ± 13	40 ± 13
BMI (R)	29 ± 5	29 ± 5	29 ± 6	29 ± 5	28 ± 5
BMI (D) Baseline	29 ± 7	29 ± 7	29 ± 7	28 ± 6	29 ± 7
Creatinine (R)	6 ± 3	2 ± 2	3 ± 2	2 ± 1	2 ± 1
Creatinine (D) Baseline	1 ± 1	1 ± 1	1 ± 2	1 ± 1	1 ± 1
Gender (R)					
Male	196 ± 5	222 ± 7	512 ± 9	380 ± 0	272 ± 7
Female	102 ± 5	140 ± 7	316 ± 9	237 ± 0	180 ± 7
Gender (D)					
Male	188 ± 5	214 ± 6	497 ± 10	387 ± 0	281 ± 7
Female	110 ± 5	148 ± 6	331 ± 10	230 ± 0	171 ± 7
Race					
White	192 ± 6	197 ± 7	460 ± 11	325 ± 2	209 ± 7
Black	88 ± 5	144 ± 6	315 ± 10	246 ± 2	208 ± 7
Asian	18 ± 3	21 ± 4	53 ± 6	46 ± 1	35 ± 4

* (D) Baseline means values before transplantation.

### 3.2 Prediction of short-term graft failure within the first three months

**Figure 2:**
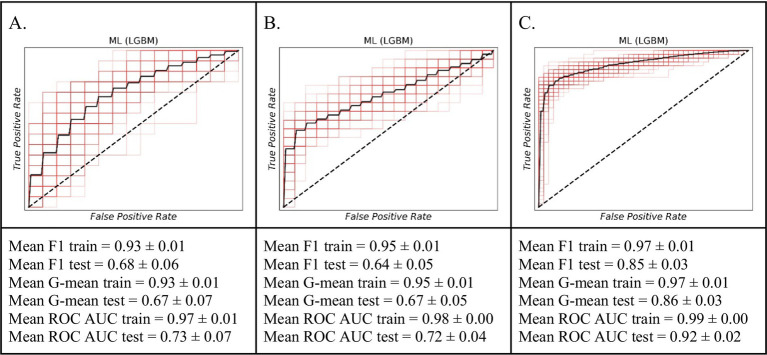
The ROC curves of the machine learning model (LGBM) for predicting short-term graft failure. **(A)** The 0–3 month period. **(B)** For the 3–9 month period. **(C)** The 9–15 month period.

In this study, we compared the predictive outcomes of the classical Cox PHR with the three ML algorithms. Figure 2A illustrates that the among ML model, LGBM classifier achieved the better prediction accuracy of graft failure during the 0–3-month period. For the training set, the model showed strong performance with a Mean ROC AUC of 0.97 ± 0.01, a Mean F1 score of 0.93 ± 0.01, and a Mean G-mean of 0.93 ± 0.01. On the test set, the model achieved a Mean ROC AUC of 0.73 ± 0.07, a Mean F1 score of 0.68 ± 0.06, and a Mean G-mean of 0.67 ± 0.07.

### 3.3 Prediction of short-term graft failure risk within a 3- to 9-month timeframe

Similarly, within 3 months, our ML model LGBM classifier exhibited considerable proficiency in predicting graft failure within the 3–9-month timeframe than other models (Figure 2B). Performance on the training data was robust, with an average F1 score of 0.95 ± 0.01, an average G-mean of 0.95 ± 0.01, and an average ROC AUC of 0.98 ± 0.00. When evaluated on external validational test data, the model’s predictive ability remained robust, yielding an average F1 score of 0.64 ± 0.05, an average G-mean of 0.67 ± 0.05, and an average ROC AUC of 0.72 ± 0.04.

### 3.4 Prediction of short-term graft failure risk within a 9- to 15-month timeframe

The ML model particularly LGBM classifier distinguished itself in accurately forecasting graft failure across the 9–15-month period (Figure 2C). Its performance on the training data was exceptional, marked by an average F1 score of 0.97 ± 0.01, an average G-mean of 0.97 ± 0.01, and an average ROC AUC of 0.99 ± 0.00. On the independent test set, the model continued to show strong predictive power, achieving an average F1 score of 0.85 ± 0.03, an average G-mean of 0.86 ± 0.03, and an average ROC AUC of 0.92 ± 0.02. These outcomes highlight the enhanced predictive power of ML models for predicting of kidney graft failure within this specific timeframe.

### 3.5 Prediction of mid-term graft failure risk within a 15- to 39-month timeframe

**Figure 3:**
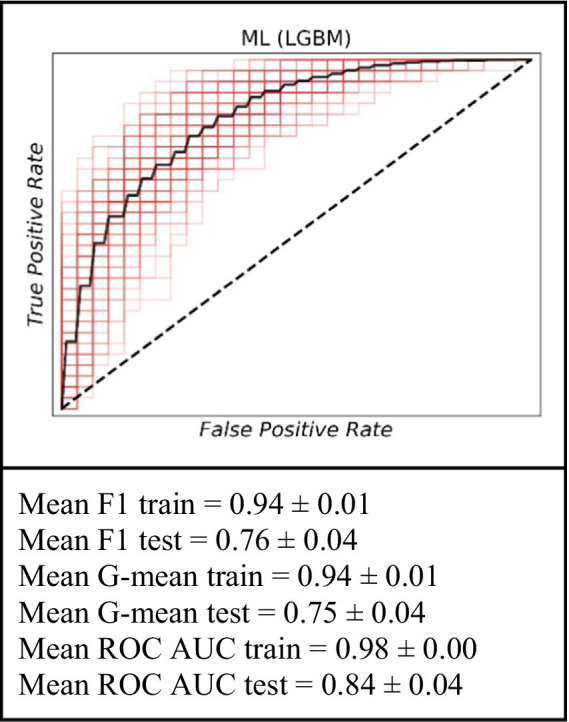
The ROC curve of the machine learning model (LGBM) for the prediction of graft failure for mid-term (for the 15–39 months).

To identify baseline relationships between variables spanning an interval from 15 to 39 months, we implemented a dynamic patient monitoring strategy. This dynamic prognostic approach demonstrated high performance in predicting graft failure using the ML model (Figure 3). Specifically, on the training dataset, the model achieved a mean F1 value of 0.94 ± 0.01, a mean G value of 0.94 ± 0.01, and a mean ROC AUC of 0.98 ± 0.00. For the testing set, the model achieved a mean F1 value of 0.76 ± 0.04, a mean G value of 0.75 ± 0.04, and a mean ROC AUC of 0.84 ± 0.04. The ML model demonstrated superior prognostic value and the inclusion of dynamic data significantly improved the prediction accuracy for up to 3 years.

### 3.6 Prediction of long-term graft failure risk within a 39- to 72-month timeframe

**Figure 4:**
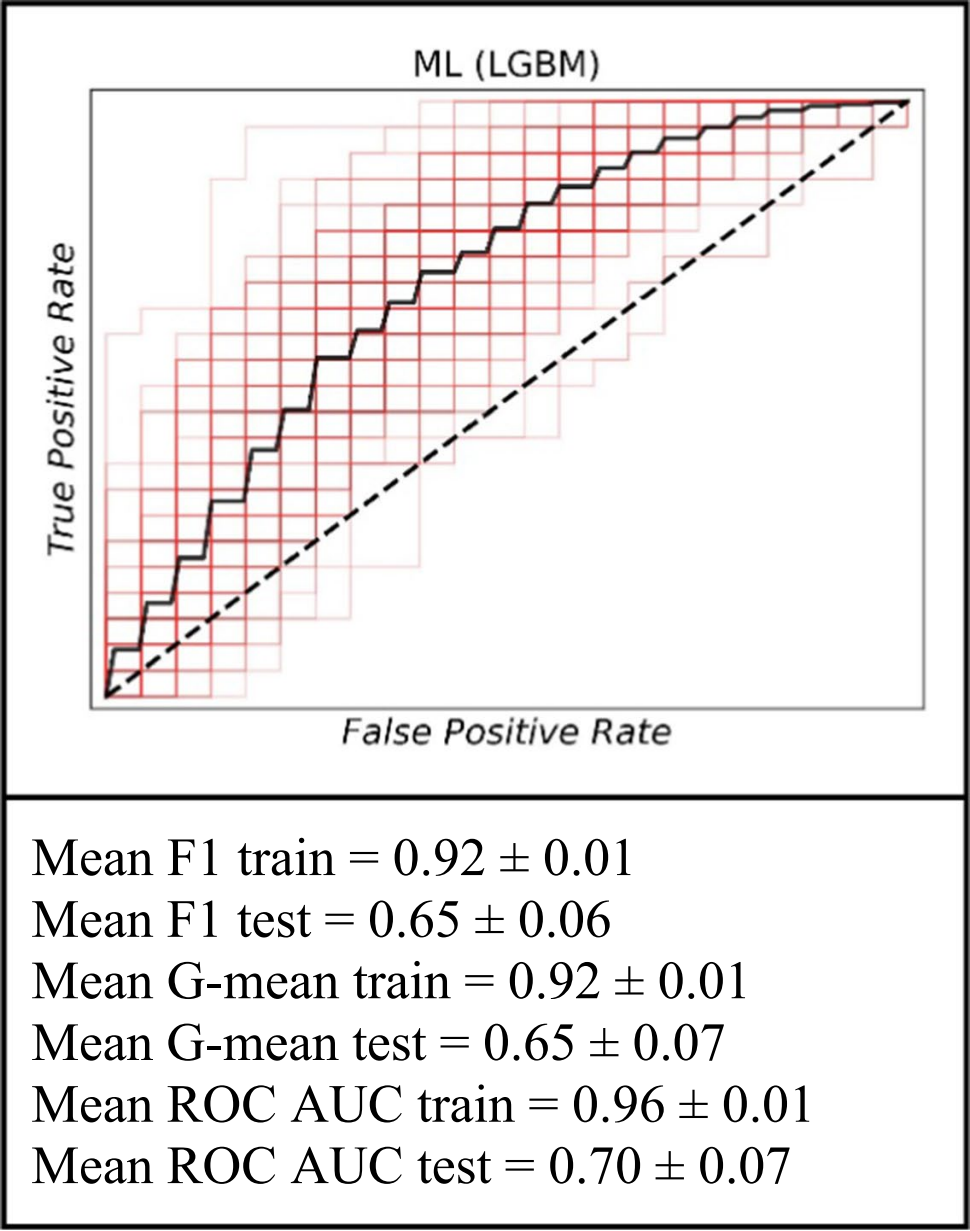
The ROC curve of the machine learning model (LGBM) for the prediction of graft failure for long-term (for the 39–72 months).

For the analysis covering the period within a 39- to 72-month timeframe, the ML model continued to exhibit its predictive capabilities. On the training data, the model achieved an average F1 score of 0.92 ± 0.01, an average G-mean of 0.92 ± 0.01, and an average ROC AUC of 0.96 ± 0.01. When evaluated on the test set, the performance was marked by an average F1 score of 0.65 ± 0.06, an average G-mean of 0.65 ± 0.07, and an average ROC AUC of 0.70 ± 0.07. These results further support the utility of the ML approach in long-term graft failure prediction (Figure 4).

## 4 Discussion

This study demonstrates the utility of ML algorithms, particularly the Light Gradient Boosting Machine (LGBM) model for predicting phase-specific graft failure following deceased donor kidney transplantation. By utilizing clinically relevant donor and recipient data from a 2015–2020 cohort and stratifying outcomes across five discrete post-transplant intervals, our findings support the integration of ML models into transplant decision-making frameworks, particularly when dynamic monitoring and phase-specific risk assessment are required.

The LGBM model demonstarted relatively improved prediction of graft failure across all timeframes among Ml and traditional statistcial model, with especially high accuracy in the 9–15-month and 15–39-month intervals, where AUCs reached 0.92 and 0.84 on the test sets, respectively. These results are in line with previous findings that demonstrate the advantages of gradient boosting algorithms in capturing complex, nonlinear interactions between clinical variables (Huang et al., 2022; Raynaud et al., 2021). The peak performance observed in the 9–15-month interval may be attributed to the availability of informative early post-transplant data that are predictive of subclinical or emerging chronic injury. During this period, patients often experience immunologic changes, subtle declines in graft function, or delayed complications, all of which may be imperceptible to traditional linear models but readily learnable by ML classifiers. We observed that model performance was substantially boosted when incorporating dynamic post-transplant data. The inclusion of follow-up clinical markers at 1 year and 2 years post-transplant, led to the highest predictive accuracy in the mid-term periods. Specifically, the model’s performance peaked in the 9–15-month window (test AUC ~ 0.92, F1 ~ 0.85), and remained high for the 15–39-month window (AUC ~ 0.84 on the test set). In contrast, prediction of graft failure based solely on baseline variables (e.g., at transplantation for 0–3 months, or even projecting out to 5 years without interim updates) was less accurate (test AUC in the ~0.65–0.73 range). This pattern underscores the value of updating risk predictions with intermediate clinical data. In essence, our findings support a dynamic prognostic strategy: by integrating time-updated patient information, the model can more effectively identify patients at risk of graft failure up to 3 years post-transplant. This result is consistent with prior evidence that incorporating post-transplant variables (such as the patient’s serum creatinine at 3–12 months) markedly improves long-term graft survival predictions (Yoo et al., 2017; Lenain et al., 2021).

The performance of our model aligns with, and in some aspects exceeds, results reported in previous studies on graft outcome prediction. A recent systematic review and meta-analysis of 27 studies found that ML models achieved an overall mean AUC of approximately 0.82 for predicting kidney graft survival (Ravindhran et al., 2023). In our work, the mid-term prognostic models (particularly the 9–15-month risk model) surpassed this benchmark, achieving an AUC above 0.90, while even our longer-term model (predicting 5–6-year failure risk) attained a comparable discrimination (~0.70–0.72) to other state-of-the-art approaches (Lenain et al., 2021). These findings underscore that our ML framework is competitive with the best-performing models in the literature. Moreover, our results extend and strengthen the growing body of evidence favoring ML-based approaches in transplant outcome modeling. While conventional Cox models offer transparency and interpretability, their assumption of linearity and constant hazard ratios over time limits their utility in dynamic clinical contexts. Prior studies have reported modest performance for static models, with C-indices 0.55 when applied to long-term graft outcomes (Huang et al., 2022). In contrast, our LGBM-based model achieved comparable or higher performance with enhanced adaptability across timeframes.

**Figure 5:**
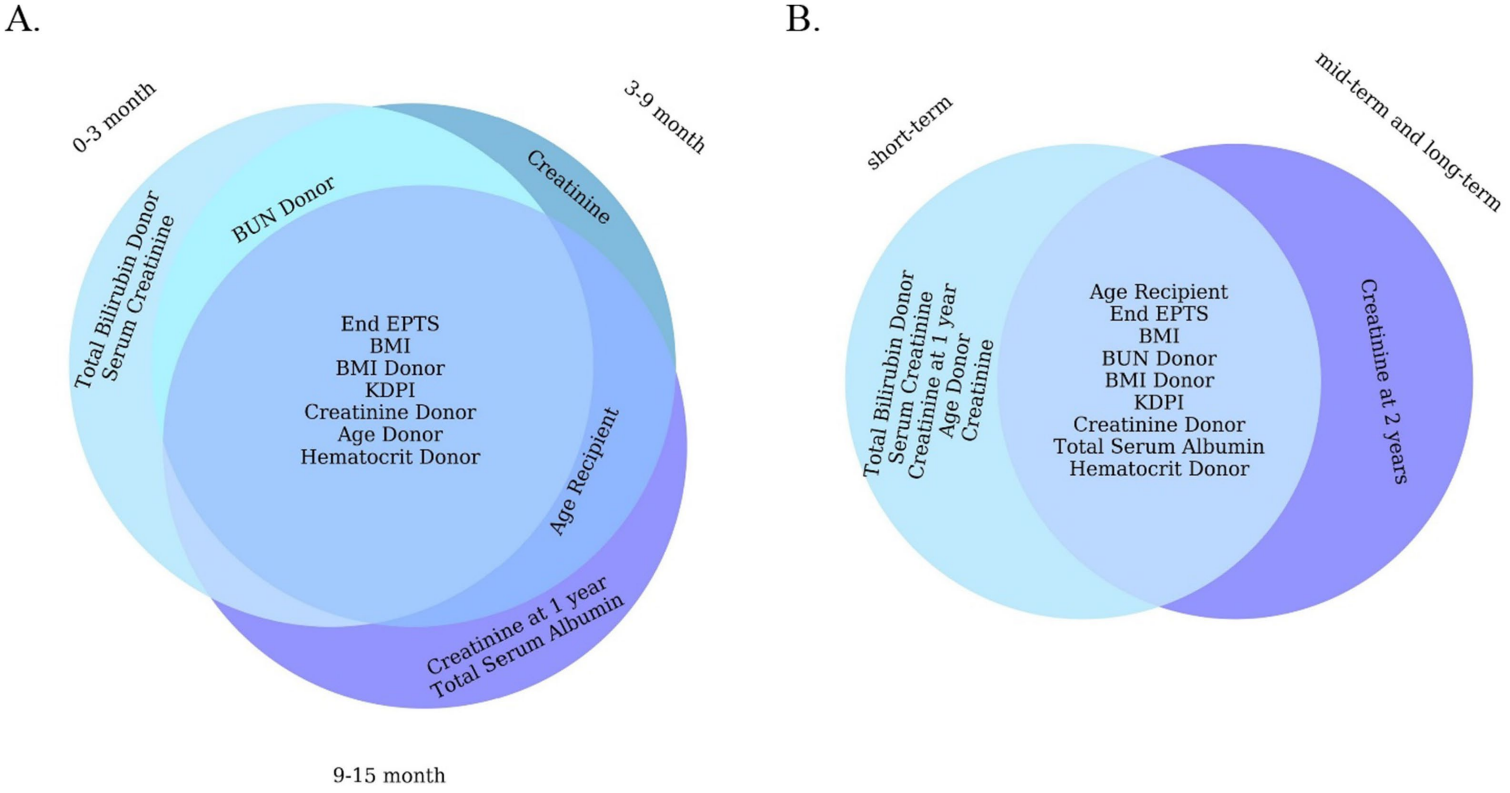
Venn diagram of 10 important features of a machine learning model (LGBM). **(A)** For short-term. **(B)** For all short-term, mid-term, and long-term.

Beyond raw performance metrics, an important aspect of our analysis is the clinical relevance of the predictors identified by the ML models. The features with highest importance in our models (Figure 5) are largely consistent with known risk factors for graft loss in majority case the early and late post-transplant periods, the best-performing LGBM model identified key predictors of graft failure were serum creatinine levels, the body mass index, Kidney Donor Profile Index (KDPI), Estimated Post-Transplant Survival (EPTS) score, donor/recipient age, donor bilirubin, donor hematocrit and blood urea nitrogen. Among these, serum creatinine emerged as a particularly significant biomarker. It is routinely utilized in clinical practice as an indicator of renal function and serves as a reliable marker for monitoring allograft health (Josephson, 2011). Although elevated serum creatinine levels typically reflect impaired kidney function, individuals with higher pre-transplant serum creatinine, which is often indicative of greater muscle mass, have been associated with improved graft and patient survival outcomes following transplantation (Streja et al., 2011). Interestingly, the KDPI and EPTS had a strong predictive power in ML models in all five timeframes. A lower percentage in both the KDPI and the EPTS score is indicative of a longer anticipated post-transplant survival, while higher percentage scores are associated with reduced post-transplant survival (Prunster et al., 2023; EPTS, 2020). For example, a KDPI score of 0% reflects a donor kidney with superior predicted allograft survival compared to all other donor kidneys transplanted within the same calendar year. Conversely, a KDPI score of 100% denotes a kidney with the poorest expected allograft survival relative to other kidneys transplanted during that period (KDPI, 2020). Previous research has suggested that individuals of Black race may have a higher susceptibility to graft failure following kidney transplantation compared to White individuals (Kwan et al., 2018; Becerra et al., 2022; DiFranza et al., 2024). However, there is a notable paucity of studies that concurrently examine all three major ethnic groups; to our knowledge, this study is the first to conduct a comparative analysis across these three populations. Future research should prioritize the inclusion of diverse populations to ensure fairness and the broader applicability of predictive models for all patient groups.

This study is not without limitations. The retrospective design, although valuable for model development, inherently introduces bias and limits causal inference. A notable limitation of our study is the absence of formal clinical impact evaluation metrics, such as decision curve analysis or clinical impact curves. While our phase-based models demonstrated strong predictive performance, we did not assess their net clinical benefit or decision-making utility in practice. Future work will incorporate these methods, particularly during external validation, to more rigorously quantify the real-world clinical applicability of our models. Another limitation is the absence of longitudinal biomarker, immunologic, or histopathological data, which are increasingly recognized as potent predictors of graft survival (Raynaud et al., 2021; Zhunussov et al., 2025). Future research should focus on integrating temporal biomarker dynamics, histopathology, and genomics into model training pipelines to enhance long-term predictive validity. Additionally, deployment in prospective clinical settings with real-time data ingestion and clinician feedback will be essential for assessing the real-world utility and trustworthiness of these AI-assisted models. It’s crucial to acknowledge that the decision to exclude patients from ethnic minority groups from the primary cohort study warrants careful ethical consideration (Salybekov et al., 2025). Such exclusion could limit the generalizability of the findings and potentially exacerbate existing healthcare disparities. One of our main limitations is that we have not yet confirmed how well this model will work in practice in a clinical setting. This will be a major focus of the next phase of the study.

In conclusion, this discussion highlights that our ML-based predictive model, validated on multi-year and multi-ethnic transplant cohorts, offers a sophisticated and evidence-backed tool for forecasting kidney graft outcomes. It aligns with and builds upon findings in the literature: dynamic, data-driven predictions can significantly outperform static models (Huang et al., 2022) and provide actionable insights to guide patient management (Raynaud et al., 2021). By stratifying risk from the early postoperative period through the mid- to long-term, such models enable a proactive approach to allograft care. With ongoing refinement and integration into clinical workflows, predictive analytics in transplantation has the potential to extend graft survival and ultimately improve the lives of kidney transplant recipients.

## Data Availability

The original contributions presented in the study are included in the article/Supplementary material, further inquiries can be directed to the corresponding authors.

## Footnotes

^1^ https://www.Python.Org
