# Supplementary material for: Phase-specific kidney graft failure prediction with machine learning model
Source: Front Artif Intell. 2025 Oct 2;8:1682639. doi: 10.3389/frai.2025.1682639 (PMC12528114; doi:10.3389/frai.2025.1682639)
Supplement: Supplementary file 1 [file Data_Sheet_1.DOCX]

Supplementary Material

# Supplementary Figures and Tables

## Supplementary Figures

| A.  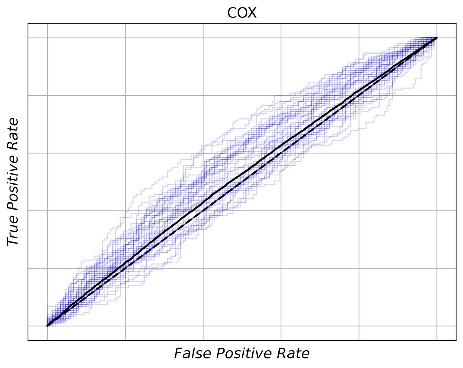 | B.  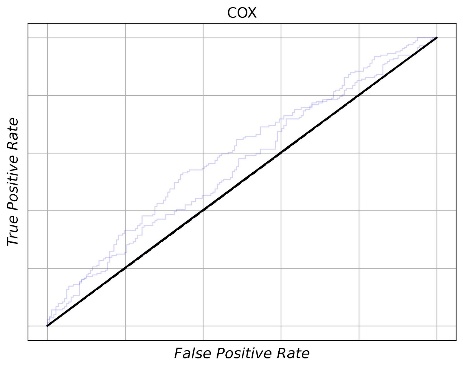 | C.  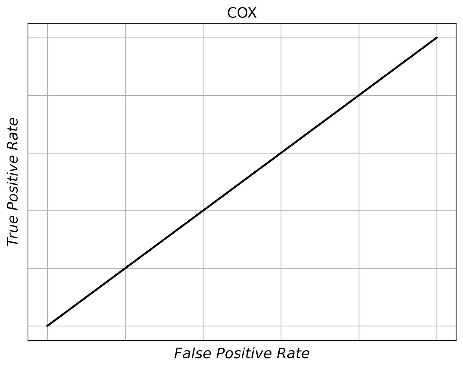 |  |
| --- | --- | --- | --- |
|  |  |  |  |
| Mean ROC AUC = 0.52 ± 0.04  Mean C-index = 0.52 ± 0.03 | Mean ROC AUC = 0.50 ± 0.01  Mean C-index = 0.50 ± 0.01 | Mean ROC AUC = 0.50 ± 0.00  Mean C-index = 0.50 ± 0.00 |  |

**Supplementary Figure 1.** The ROC curves of the Cox model for the prediction of short-term graft failure. A. The 0-3 month period. B. For the 3-9 month period. C. The 9-15-month period

| 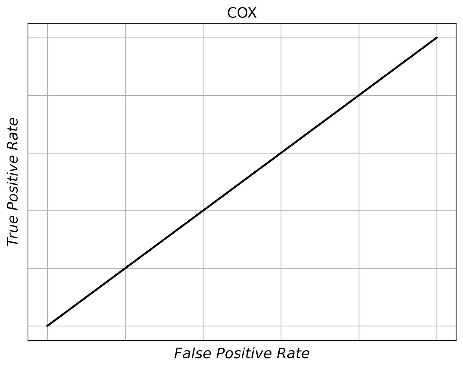 |
| --- |
| Mean ROC AUC = 0.50 ± 0.00  Mean C-index = 0.50 ± 0.00 |

**Supplementary Figure 2.** The ROC curve of the Cox model for the prediction of graft failure for mid-term (for the 15-39 months)

| 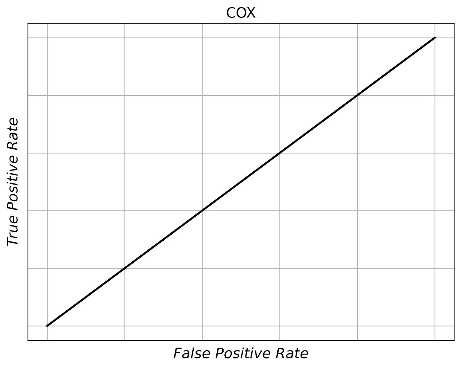 |
| --- |
| Mean ROC AUC = 0.50 ± 0.00  Mean C-index = 0.50 ± 0.00 |

**Supplementary Figure 3.** The ROC curve of the Cox model for the prediction of graft failure for long-term (for the 39-72 months)

| A.  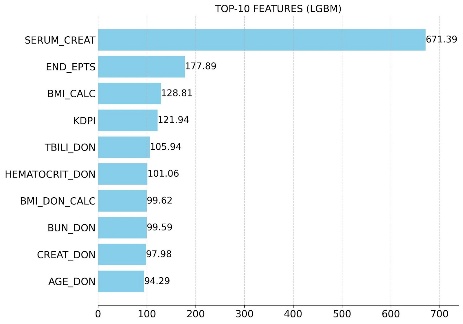 | B.  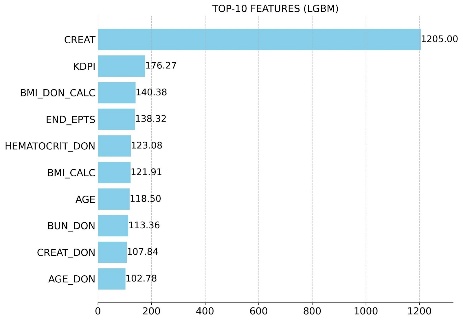 | C.  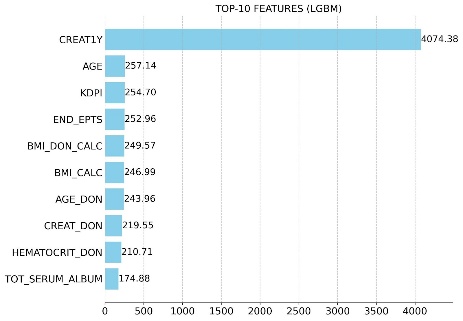 |  |
| --- | --- | --- | --- |
|  |  |  |  |

**Supplementary Figure 4.** Top 10 important features of the machine learning (LGBM) model for predicting short-term graft failure. A. Features relevant for the 0-3 month prediction period. B. Features relevant for the 3-9 month prediction period. C. Features relevant for the 9-15 month prediction period


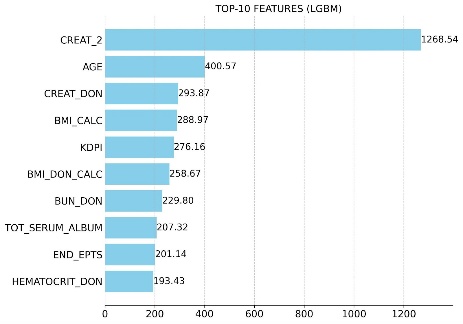


**Supplementary Figure 5.** Top 10 important features of the machine learning (LGBM) model for predicting mid-term graft failure (for the 15-39 months)


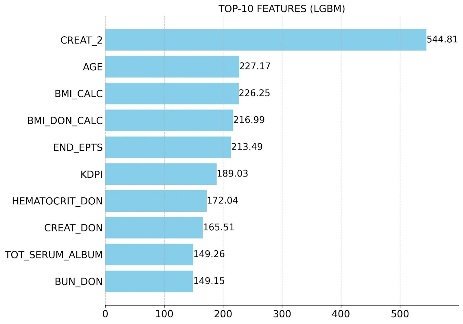


**Supplementary Figure 6.** Top 10 important features of the machine learning (LGBM) model for predicting long-term graft failure (for the 39-72 months)

| A.  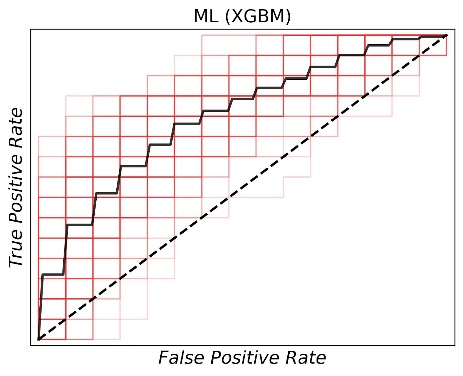 | B.  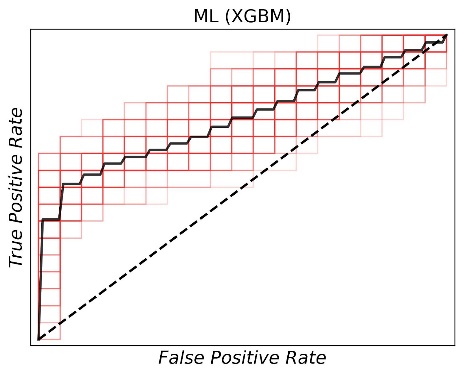 | C.  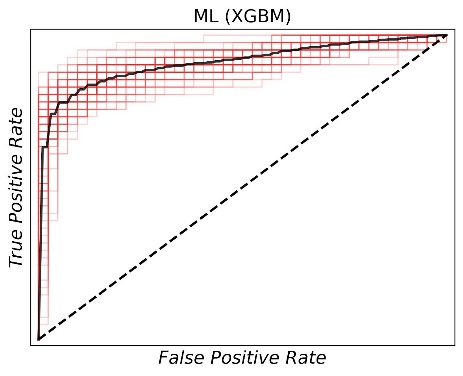 |  |
| --- | --- | --- | --- |
|  |  |  |  |
| Mean F1 train = 0.93 ± 0.01  Mean F1 test = 0.68 ± 0.07  Mean G-mean train = 0.93 ± 0.01  Mean G-mean test = 0.67 ± 0.07  Mean ROC AUC train = 0.97 ± 0.01  Mean ROC AUC test = 0.73 ± 0.07 | Mean F1 train = 0.95 ± 0.01  Mean F1 test = 0.65 ± 0.05  Mean G-mean train = 0.95 ± 0.01  Mean G-mean test = 0.68 ± 0.05  Mean ROC AUC train = 0.98 ± 0.01  Mean ROC AUC test = 0.73 ± 0.04 | Mean F1 train = 0.94 ± 0.01  Mean F1 test = 0.85 ± 0.03  Mean G-mean train = 0.94 ± 0.01  Mean G-mean test = 0.86 ± 0.03  Mean ROC AUC train = 0.98 ± 0.00  Mean ROC AUC test = 0.91 ± 0.02 |  |

**Supplementary Figure 7.** The ROC curves of the machine learning model (XGBM) for predicting short-term graft failure. A. The 0-3 month period. B. For the 3-9 month period. C. The 9-15-month period

| 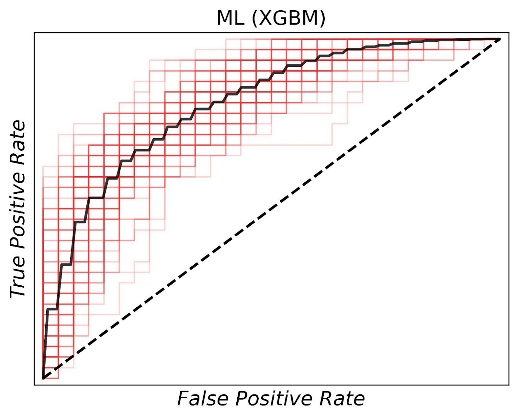 |
| --- |
| Mean F1 train = 0.90 ± 0.01  Mean F1 test = 0.74 ± 0.04  Mean G-mean train = 0.90 ± 0.01  Mean G-mean test = 0.73 ± 0.05  Mean ROC AUC train = 0.96 ± 0.00  Mean ROC AUC test = 0.81 ± 0.04 |

**Supplementary Figure 8.** The ROC curve of the machine learning model (XGBM) for the prediction of graft failure for mid-term (for the 15-39 months)

| 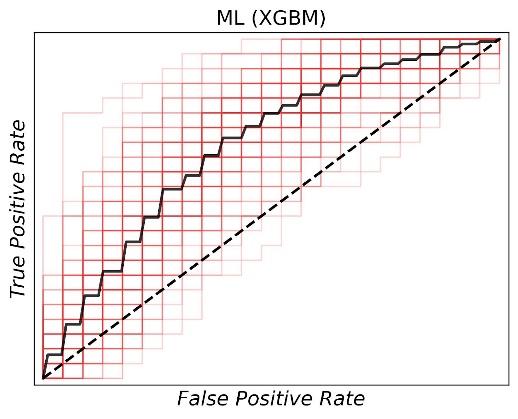 |
| --- |
| Mean F1 train = 0.90 ± 0.01  Mean F1 test = 0.64 ± 0.07  Mean G-mean train = 0.90 ± 0.01  Mean G-mean test = 0.64 ± 0.07  Mean ROC AUC train = 0.94 ± 0.01  Mean ROC AUC test = 0.69 ± 0.08 |

**Supplementary Figure 9.** The ROC curve of the machine learning model (XGBM) for the prediction of graft failure for long-term (for the 39-72 months)

| A.  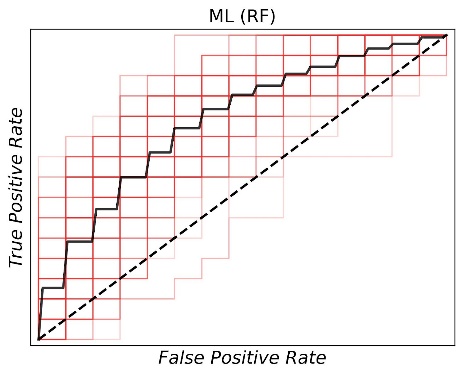 | B.  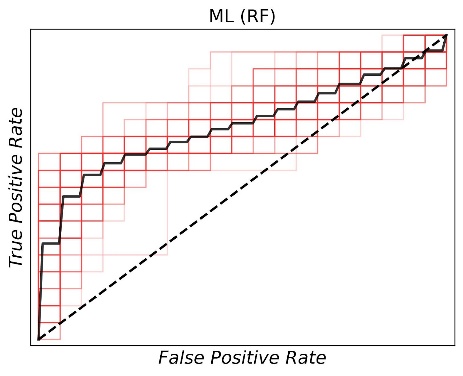 | C.  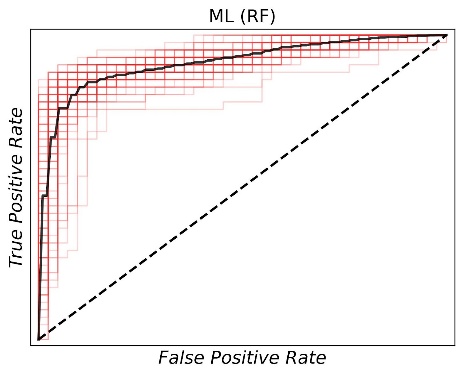 |  |
| --- | --- | --- | --- |
|  |  |  |  |
| Mean F1 train = 0.84 ± 0.01  Mean F1 test = 0.68 ± 0.06  Mean G-mean train = 0.83 ± 0.02  Mean G-mean test = 0.66 ± 0.07  Mean ROC AUC train = 0.92 ± 0.01  Mean ROC AUC test = 0.72 ± 0.08 | Mean F1 train = 0.85 ± 0.01  Mean F1 test = 0.66 ± 0.04  Mean G-mean train = 0.86 ± 0.01  Mean G-mean test = 0.69 ± 0.04  Mean ROC AUC train = 0.94 ± 0.01  Mean ROC AUC test = 0.71 ± 0.05 | Mean F1 train = 0.88 ± 0.01  Mean F1 test = 0.86 ± 0.03  Mean G-mean train = 0.88 ± 0.01  Mean G-mean test = 0.87 ± 0.03  Mean ROC AUC train = 0.96 ± 0.01  Mean ROC AUC test = 0.91 ± 0.03 |  |

**Supplementary Figure 10.** The ROC curves of the machine learning model (RF) for predicting short-term graft failure. A. The 0-3 month period. B. For the 3-9 month period. C. The 9-15-month period

| 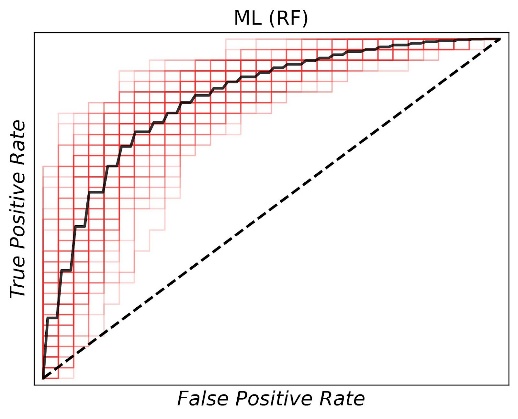 |
| --- |
| Mean F1 train = 0.82 ± 0.01  Mean F1 test = 0.77 ± 0.03  Mean G-mean train = 0.82 ± 0.01  Mean G-mean test = 0.75 ± 0.04  Mean ROC AUC train = 0.91 ± 0.01  Mean ROC AUC test = 0.83 ± 0.04 |

**Supplementary Figure 11.** The ROC curve of the machine learning model (RF) for the prediction of graft failure for mid-term (for the 15-39 months)

| 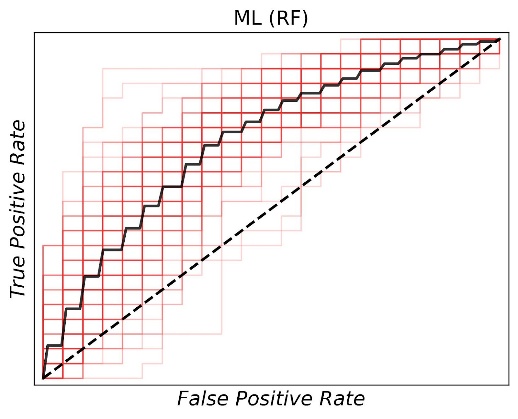 |
| --- |
| Mean F1 train = 0.80 ± 0.02  Mean F1 test = 0.66 ± 0.05  Mean G-mean train = 0.80 ± 0.02  Mean G-mean test = 0.66 ± 0.06  Mean ROC AUC train = 0.89 ± 0.01  Mean ROC AUC test = 0.70 ± 0.07 |

**Supplementary Figure 12.** The ROC curve of the machine learning model (RF) for the prediction of graft failure for long-term (for the 39-72 months)

**Supplementary Table 1. TRIPOD+AI checklist**

| **Section/Topic Item Development Checklist item**  **/ evaluation** | | | | **Reported on page** | **Text Excerpt** | |
| --- | --- | --- | --- | --- | --- | --- |
| **TITLE** | | | |  |  | |
| *Title* | 1 | D;E | Identify the study as developing or evaluating the performance of a multivariable prediction model, the target population, and the outcome to be predicted | 1 | “Phase-Specific Kidney Graft Failure Prediction with Machine Learning Model” | |
| **ABSTRACT** | | | | |  | |
| *Abstract* | 2 | D;E | See TRIPOD+AI for Abstracts checklist | 1 | BACKGROUND/OBJECTIVE: “ Accurate prediction of kidney graft failure […] present a promising alternative”  METHODS: “ This study developed and dynamically evaluated […] using ROC AUC, F1 score, and G-mean”  RESULTS: “ The ML models demonstrated varying performance across […] Long-term prediction from 39–72 months was more challenging (ROC AUC = 0.70 ± 0.07; F1 score = 0.65 ± 0.06).”  CONCLUSION: “ Phase-specific ML models offer robust predictive performance […] long-term transplant outcomes.” | |
| **INTRODUCTION** | | | | |  | |
| *Background* | 3a | D;E | Explain the healthcare context (including whether diagnostic or prognostic) and rationale for developing or evaluating the prediction model, including references to existing models | 2 | “Chronic kidney diseases (CKD) affect […] risk estimates in patient data are necessary.” | |
|  | 3b | D;E | Describe the target population and the intended purpose of the prediction model in the context of the care pathway, including its intended users (e.g., healthcare professionals, patients, public) | 2 | “This study aimed to develop and dynamically assess phase-specific machine learning models […] post-transplant intervals.”  “[…] remains widely utilized due to its robustness, reliability, and interpretability for clinicians.”  “[…] ML-based models outperform traditional Cox models in discrimination and overall accuracy for graft survival prediction.” | |
|  | 3c | D;E | Describe any known health inequalities between sociodemographic groups | 2 | “However, in many countries, there is a shortage of renal replacement and kidney transplantation services, and an estimated 2.3–7.1 million adults have died prematurely from lack of access to this treatment.” | |
| *Objectives* | 4 | D;E | Specify the study objectives, including whether the study describes the development or validation of a prediction model (or both) | 2 | “This study aimed to develop and dynamically assess phase-specific machine learning models […] post-transplant intervals.” | |
| **METHODS** | | | | |  | |
| *Data* | 5a | D;E | Describe the sources of data separately for the development and evaluation datasets (e.g., randomised trial, cohort, routine care or registry data), the rationale for using these data, and representativeness of the data | 3 | This study is predicated upon data derived from the United States National Kidney Transplantation Database (UNOS/OPTN) […] and follow-up data from recipients. | |
|  | 5b | D;E | Specify the dates of the collected participant data, including start and end of participant accrual; and, if applicable, end of follow-up | 3 | “This study is predicated upon data derived from the United States National Kidney Transplantation Database (UNOS/OPTN), spanning the years 2015 to 2021.” | |
| *Participants* | 6a | D;E | Specify key elements of the study setting (e.g., primary care, secondary care, general population)  including the number and location of centres | 3 | “Selected transplant recipients and donors were between 18 to 80 years old and had undergone a primary kidney transplantation.” | |
|  | 6b | D;E | Describe the eligibility criteria for study participants | 3 | “Selected transplant recipients and donors were between 18 to 80 years old […] treatment effectiveness and complication risk factors.” | |
|  | 6c | D;E | Give details of any treatments received, and how they were handled during model development or evaluation, if relevant | 3-4 | “Essentially, this methodology serves as a form of dynamic prediction, allowing for the continuous adjustment of prognostic models in real time by accounting for patient condition fluctuations.”  “For the data analysis, we included pre-operative patients' and donors' data (waiting list records), and follow-up data from recipients. ” | |
| *Data preparation* | 7 | D;E | Describe any data pre-processing and quality checking, including whether this was similar across  relevant sociodemographic groups | 3 | “Considering the STROBE guidelines, the study design aimed to ensure transparency and completeness in reporting the results.” | |
| *Outcome* | 8a | D;E | Clearly define the outcome that is being predicted and the time horizon, including how and when assessed, the rationale for choosing this outcome, and whether the method of outcome assessment is  consistent across sociodemographic groups | 3 | “The dataset was stratified into five distinct cohorts […] records per subject were included.” | |
|  | 8b | D;E | If outcome assessment requires subjective interpretation, describe the qualifications and demographic characteristics of the outcome assessors | 3 | “This multi-stage analytical approach enabled […] for patient condition fluctuations.” | |
|  | 8c | D;E | Report any actions to blind assessment of the outcome to be predicted | 4 | “The dataset was initially partitioned into training and testing subsets, with 10% reserved for testing […] StratifiedKFold […].” | |
| *Predictors* | 9a | D | Describe the choice of initial predictors (e.g., literature, previous models, all available predictors) and  any pre-selection of predictors before model building | 3-4 | “Selected transplant recipients and donors were between 18 to 80 years old and had undergone a primary kidney transplantation. In our study, we set inclusion and exclusion criteria to minimize potential biases and ensure the analysis was conducted on a cohort most representative of the target patient population […].”  “For the data analysis, we included pre-operative patients' and donors' data (waiting list records), and follow-up data from recipients […].”  “Subsequently, feature selection was performed on the combined train+validation set using the three commonly used ML algorithms, namely RF (Random Forest), XGB (XGBoost), and LGBM (LightGBM) […].” | |
|  | 9b | D;E | Clearly define all predictors, including how and when they were measured (and any actions to blind assessment of predictors for the outcome and other predictors) | 3 | “For the data analysis, we included pre-operative patients' and donors' data (waiting list records), and follow-up data from recipients […].”  “The dataset was stratified into five distinct cohorts based on post-transplantation preliminary statistical findings as 0–3, 3–9, 9–15, 15–39, and 39–72 months […].” | |
|  | 9c | D;E | If predictor measurement requires subjective interpretation, describe the qualifications and demographic characteristics of the predictor assessors | 3 | “[…] we included pre-operative patients' and donors' data (waiting list records), and follow-up data from recipients […].” | |
| *Sample size* | 10 | D;E | Explain how the study size was arrived at (separately for development and evaluation), and justify that  the study size was sufficient to answer the research question. Include details of any sample size calculation | 4 | “The baseline characteristics of the study cohorts were stratified across five-time intervals: 0–3 months (N=298), 3–9 months (N=362), 9–15 months (N=828), 15–39 months (N=617), and 39–72 months (N=452).” | |
| *Missing data* | 11 | D;E | Describe how missing data were handled. Provide reasons for omitting any data | 3 | “Prior to imputation, features with more than 30% missing values were removed. The imputation of missing values are performed via multiple imputation chained equations (MICE) using the *miceforest* package.” | |
| *Analytical methods* | 12a | D | Describe how the data were used (e.g., for development and evaluation of model performance) in the analysis, including whether the data were partitioned, considering any sample size requirements | 3-4 | “The dataset was initially partitioned into training and testing subsets, with 10% reserved for testing. This ensures unbiased evaluation. The remaining training set underwent stratified 5-fold cross-validation (StratifiedKFold).” | |
|  | 12b | D | Depending on the type of model, describe how predictors were handled in the analyses (functional form,  rescaling, transformation, or any standardisation). | 3 | “Undersampling was conducted to control data imbalance […] classification was subsequently considered during result interpretation.” | |
|  | 12c | D | Specify the type of model, rationale^2^, all model-building steps, including any hyperparameter tuning,  and method for internal validation | 3-4 | “Subsequently, feature selection was performed on the combined train […] the GridSearchCV approach with fivefold cross-validation.” | |
|  | 12d | D;E | Describe if and how any heterogeneity in estimates of model parameter values and model performance was handled and quantified across clusters (e.g., hospitals, countries). See TRIPOD-Cluster for  additional considerations^3^ | 3-4 | “The dataset was stratified into five distinct cohorts […] accounting for patient condition fluctuations.”  “Second, a standalone Cox Proportional Hazards Regression (Cox PHR) model was implemented as a baseline for time-to-event analysis.” | |
|  | 12e | D;E | Specify all measures and plots used (and their rationale) to evaluate model performance (e.g., discrimination, calibration, clinical utility) and, if relevant, to compare multiple models | 4 | “The mean ROC-AUC served as the primary performance metric, ensuring robust and reliable model evaluation. In addition, F1 and G-Mean scores were employed to assess the overall predictive capability of the trained models.” | |
|  | 12f | E | Describe any model updating (e.g., recalibration) arising from the model evaluation, either overall or for particular sociodemographic groups or settings | 3 | “[…] we included pre-operative patients' and donors' data (waiting list records), and follow-up data from recipients […].” | |
|  | 12g | E | For model evaluation, describe how the model predictions were calculated (e.g., formula, code, object, application programming interface) | 4 | “The mean ROC-AUC served as the primary performance metric, ensuring robust and reliable model evaluation. In addition, F1 and G-Mean scores were employed to assess the overall predictive capability of the trained models.” | |
| *Class imbalance* | 13 | D;E | If class imbalance methods were used, state why and how this was done, and any subsequent methods to  recalibrate the model or the model predictions | 3 | “Undersampling was conducted to control data imbalance, such as the number of observations in the majority class was reduced to match that of the minority class through RandomUnderSampling. The iteration rate was set to 100 repetitions.” | |
| *Fairness* | 14 | D;E | Describe any approaches that were used to address model fairness and their rationale | 3 | “Undersampling was conducted to control data imbalance […].”  “Patients from ethnic minority groups [...] were excluded […].” | |
| *Model output* | 15 | D | Specify the output of the prediction model (e.g., probabilities, classification). Provide details and  rationale for any classification and how the thresholds were identified | 4 | “The mean ROC-AUC served as the primary performance metric, ensuring robust and reliable model evaluation. In addition, F1 and G-Mean scores were employed to assess the overall predictive capability of the trained models.” | |
| *Training versus*  *evaluation* | 16 | D;E | Identify any differences between the development and evaluation data in healthcare setting, eligibility criteria, outcome, and predictors | 3 | “The dataset was stratified into five distinct cohorts [...] For each cohort, repetitive samples were generated by selecting data from a single randomly selected patient visit [...].” |  |
| *Ethical approval* | 17 | D;E | Name the institutional research board or ethics committee that approved the study and describe the participant-informed consent or the ethics committee waiver of informed consent | 3 | “This approach adhered to ethical guidelines for patient data utilization, ensuring privacy and responsible research practices.” |  |
| **OPEN SCIENCE** | | | | |  |  |
| *Funding* | 18a | D;E | Give the source of funding and the role of the funders for the present study | 8 | “This research was funded by JSPS KAKENHI funding # 2 3 K 1 7 0 0 2 & the Science Committee of the Ministry of Science and Higher Education of the Republic of Kazakhstan for providing grant # AP14872543.” |  |
| *Conflicts of interest* | 18b | D;E | Declare any conflicts of interest and financial disclosures for all authors | 8 | “The authors declare that the research was conducted in the absence of any commercial or financial relationships that could be construed as a potential conflict of interest.” |  |
| *Protocol* | 18c | D;E | Indicate where the study protocol can be accessed or state that a protocol was not prepared |  | NA |  |
| *Registration* | 18d | D;E | Provide registration information for the study, including register name and registration number, or state that the study was not registered |  | NA |  |
| *Data sharing* | 18e | D;E | Provide details of the availability of the study data | 7 | “The original contributions presented in the study are included in the article/supplementary material, further inquiries can be directed to the corresponding author.  The data supporting the findings of this study are subject to restrictions by the Organ Procurement and Transplantation Network (OPTN) / United Network for Organ Sharing (UNOS) and are not publicly available due to policies designed to protect patient confidentiality. Inquiries regarding access to these data should be directed to OPTN/UNOS. ” |  |
| *Code sharing* | 18f | D;E | Provide details of the availability of the analytical code |  | The code is available upon request. |  |
| **PATIENT & PUBLIC INVOLVEMENT** | | | | |  |  |
| *Patient & Public Involvement* | 19 | D;E | Provide details of any patient and public involvement during the design, conduct, reporting, interpretation, or dissemination of the study or state no involvement. |  | NA |  |
| **RESULTS** | | | | |  |  |
| *Participants* | 20a | D;E | Describe the flow of participants through the study, including the number of participants with and without the outcome and, if applicable, a summary of the follow-up time. A diagram may be helpful. | 4 | “The baseline characteristics of the study cohorts were stratified across five-time intervals: 0–3 months (N=298), 3–9 months (N=362), 9–15 months (N=828), 15–39 months (N=617), and 39–72 months (N=452).” |  |
|  | 20b | D;E | Report the characteristics overall and, where applicable, for each data source or setting, including the key dates, key predictors (including demographics), treatments received, sample size, number of outcome events, follow-up time, and amount of missing data. A table may be helpful. Report any differences across key demographic groups. | 4 | “Clinically relevant data from transplant recipients and donors […] characteristics of R and D are shown in Table 1.” |  |
|  | 20c | E | For model evaluation, show a comparison with the development data of the distribution of important predictors (demographics, predictors, and outcome). | 4 | “The mean age of recipients (R) […] characteristics of R and D are shown in Supplementary Table 1.” |  |
| *Model development* | 21 | D;E | Specify the number of participants and outcome events in each analysis (e.g., for model development, hyperparameter tuning, model evaluation) | 4 | “The baseline characteristics of the study cohorts were stratified across five-time intervals:  0–3 months (N=298),  3–9 months (N=362),  9–15 months (N=828),  15–39 months (N=617),  39–72 months (N=452).” |  |
| *Model specification* | 22 | D | Provide details of the full prediction model (e.g., formula, code, object, application programming interface) to allow predictions in new individuals and to enable third-party evaluation and implementation, including any restrictions to access or re-use (e.g., freely available, proprietary)^5^ | 4 | “In this study, we compared the predictive outcomes of the classical Cox PHR with the three ML algorithms.” |  |
| *"Model performance* | 23a | D;E | Report model performance estimates with confidence intervals, including for any key subgroups (e.g., sociodemographic). Consider plots to aid presentation. | 4-5 | “ Figure 2A illustrates that the among ML model […] and a Mean G-mean of 0.67 ± 0.07.”  “Similarly, within three months, our ML model […] and an average ROC AUC of 0.72 ± 0.04.”  “The ML model particularly LGBM classifier […] training set and 0.29 on the test set.”  “To identify baseline relationships between variables […] prediction accuracy for up to three years.”  “For the analysis covering the period […] the ML approach in long-term graft failure prediction.” |  |
|  | 23b | D;E | If examined, report results of any heterogeneity in model performance across clusters. See TRIPOD Cluster for additional details. |  | NA |  |
| *Model updating* | 24 | E | Report the results from any model updating, including the updated model and subsequent performance |  | No model updating procedures were performed in this study. |  |
| **DISCUSSION** | | | | |  |  |
| *Interpretation* | 25 | D;E | Give an overall interpretation of the main results, including issues of fairness in the context of the  objectives and previous studies | 6-7 | “This study demonstrates the utility of ML algorithms […] kidney transplantation compared to White individuals.” |  |
| *Limitations* | 26 | D;E | Discuss any limitations of the study (such as a non-representative sample, sample size, overfitting, missing data) and their effects on any biases, statistical uncertainty, and generalizability | 7 | “This study is not without limitations. […] exacerbate existing healthcare disparities.” |  |
| *Usability of the model in the context of current care* | 27a | D | Describe how poor quality or unavailable input data (e.g., predictor values) should be assessed and handled when implementing the prediction model | 6-7 | “We observed that model performance was substantially boosted when incorporating dynamic post-transplant data [...].”  “Another limitation is the absence of longitudinal biomarker, immunologic, or histopathological data [...].” |  |
|  | 27b | D | Specify whether users will be required to interact in the handling of the input data or use of the model, and what level of expertise is required of users | 6-7 | “[...] our findings support the integration of ML models into transplant decision-making frameworks [...].”  “[...] deployment in prospective clinical settings with real-time data ingestion and clinician feedback will be essential [...].” |  |
|  | 27c | D;E | Discuss any next steps for future research, with a specific view to applicability and generalizability of the model | 6-7 | “This study is not without limitations. The retrospective design, although valuable for model development, inherently introduces bias and limits causal inference[...].”  “[...] the generalizability to diverse healthcare systems or populations remains to be further validated through prospective multicenter studies.”  “Future research should focus on integrating temporal biomarker dynamics, histopathology, and genomics into model training pipelines to enhance long-term predictive validity.”  “Additionally, deployment in prospective clinical settings with real-time data ingestion and clinician feedback will be essential for assessing the real-world utility and trustworthiness of these AI-assisted models.”  “One of our main limitations is that we have not yet confirmed how well this model will work in practice in a clinical setting. This will be a major focus of the next phase of the study.” |  |
